# Emerging Promise of Cannabinoids for the Management of Pain and Associated Neuropathological Alterations in Alzheimer’s Disease

**DOI:** 10.3389/fphar.2020.01097

**Published:** 2020-07-22

**Authors:** Md. Sahab Uddin, Abdullah Al Mamun, Dewan Md. Sumsuzzman, Ghulam Md Ashraf, Asma Perveen, Simona G. Bungau, Shaker A. Mousa, Hesham R. El-Seedi, May N. Bin-Jumah, Mohamed M. Abdel-Daim

**Affiliations:** ^1^ Department of Pharmacy, Southeast University, Dhaka, Bangladesh; ^2^ Pharmakon Neuroscience Research Network, Dhaka, Bangladesh; ^3^ King Fahd Medical Research Center, King Abdulaziz University, Jeddah, Saudi Arabia; ^4^ Department of Medical Laboratory Technology, Faculty of Applied Medical Sciences, King Abdulaziz University, Jeddah, Saudi Arabia; ^5^ Glocal School of Life Sciences, Glocal University, Saharanpur, India; ^6^ Department of Pharmacy, Faculty of Medicine and Pharmacy, University of Oradea, Oradea, Romania; ^7^ Pharmaceutical Research Institute, Albany College of Pharmacy and Health Sciences, New York, NY, United States; ^8^ Division of Pharmacognosy, Department of Medicinal Chemistry, Uppsala University, Uppsala, Sweden; ^9^ Department of Chemistry, Faculty of Science, Menoufia University, Shebin El-Koom, Egypt; ^10^ Department of Biology, College of Science, Princess Nourah bint Abdulrahman University, Riyadh, Saudi Arabia; ^11^ Department of Zoology, College of Science, King Saud University, Riyadh, Saudi Arabia; ^12^ Pharmacology Department, Faculty of Veterinary Medicine, Suez Canal University, Ismailia, Egypt

**Keywords:** cannabinoids, marijuana, endocannabinoid system, pain, Alzheimer’s disease

## Abstract

Alzheimer’s disease (AD) is an irreversible chronic neurodegenerative disorder that occurs when neurons in the brain degenerate and die. Pain frequently arises in older patients with neurodegenerative diseases including AD. However, the presence of pain in older people is usually overlooked with cognitive dysfunctions. Most of the times dementia patients experience moderate to severe pain but the development of severe cognitive dysfunctions tremendously affects their capability to express the presence of pain. Currently, there are no effective treatments against AD that emphasize the necessity for increasing research to develop novel drugs for treating or preventing the disease process. Furthermore, the prospective therapeutic use of cannabinoids in AD has been studied for the past few years. In this regard, targeting the endocannabinoid system has considered as a probable therapeutic strategy to control several associated pathological pathways, such as mitochondrial dysfunction, excitotoxicity, oxidative stress, and neuroinflammation for the management of AD. In this review, we focus on recent studies about the role of cannabinoids for the treatment of pain and related neuropathological changes in AD.

## Introduction

Pain is a complex emotional and perceptual experience, which has sensory, cognitive, and affective dimensions (Bushnell et al., 2013; Talbot et al., 2019). There is a cortical response to nociceptive stimuli under vegetative as well as minimal conscious state, hence the pain perception appears crucial for survival and needs assessment in the absence of people with severe cognitive dysfunctions (De Tommaso et al., 2013). Neuropathological alterations that take place in dementia patients are accountable for changes in the perception of pain (Van Kooten et al., 2016). Though these changes can be common in different forms of dementia, however, scientists are trying to investigate the pain perception and processing in the patients of Alzheimer’s disease (AD), which is characterized by behavioral and cognitive impairments (Al Mamun et al., 2020b; Uddin et al., 2020a; Uddin et al., 2020l). Neuropathological hallmarks of AD are extracellular accumulations of amyloid beta (Aβ) as well as intracellular accumulations of neurofibrillary tangles (NFTs) that are comprised of hyperphosphorylation of tau (Uddin et al., 2019; Mamun et al., 2020; Uddin et al., 2020d). Furthermore, the development of Aβ plaques occurs primarily in the basal, orbitofrontal neocortex, and temporal areas of the brain and subsequently develops all over the hippocampus, diencephalon, neocortex, basal ganglia, and amygdala (Tiwari et al., 2019). Some events such as increased generation, oligomerization as well as accumulation of Aβ are the key factors at the beginning stage of AD. The noxious Aβ peptides including Aβ_40_ and Aβ_42_ are formed by the amyloid precursor protein (APP) through the cleavage by β- and γ-secretases (Kabir et al., 2020a; Uddin et al., 2020e; Uddin et al., 2020j). Moreover, APP is one of the transmembrane proteins that is folded and altered in the endoplasmic reticulum (ER) as well as transferred *via* the Golgi complex to the external membrane. It is evident that ER stress plays a crucial role in AD pathology. Various pathological events of AD such as accumulation of Aβ and tau proteins, disturbances in calcium (Ca^2+^) homeostasis, and oxidative stress might be triggered by ER stress in brains (Salminen et al., 2009; Uddin et al., 2020m). In contrast, this type of pathology could also produce ER stress and therefore exacerbate the pathogenesis of AD (Salminen et al., 2009; Uddin et al., 2020m).

The occurrence of chronic pain in AD patients was 45.8% (Van Kooten et al., 2016). Perception of pain might be neglected in AD patients as they might be unable to express their pain as well as seek attention as efficiently as their cognitively healthy peers (Cravello et al., 2019). Remarkably, pain is found more prevalently in severe dementia patients (van Kooten et al., 2017), and pain intensity is also connected positively with the severity of dementia (Scherder et al., 2008; Rajkumar et al., 2017; Whitlock et al., 2017). Although a bidirectional relationship exists between AD and chronic pain, however, the exact mechanism remains unclear. In a study by Hayashida and Obata, (2019) observed several common pathologies, such as aberrations of the noradrenergic system in the locus coeruleus (LC), microglial activation in brain regions including the frontal cortex, and raised central neuroinflammation in these areas in AD patients or the patients with chronic pain (Salter and Stevens, 2017). The neuropathological alterations that take place in the patients with AD selectively affect vital regions, which involved in the medial pain pathway, particularly the medial nuclei of the hypothalamus, cingulate, insula, and thalamus, while the brain regions involved in the lateral pain pathway are comparatively well conserved (Braak et al., 1993).

Cannabis, also called marijuana, has widely been used for therapeutic purposes throughout human history (Bridgeman and Abazia, 2017). The first use of this plant had been recorded about 5000 years ago in ancient China, where plant extracts were used for the treatment of pain and cramps (Zou and Kumar, 2018). Furthermore, the uses of cannabis have been recognized for medical purposes such as anti-inflammatory, anticonvulsant, anti-nociception, anti-emetic, and recreational use, which has mostly restricted its medical uses (Uddin et al., 2018; Vučkovic et al., 2018; Zou and Kumar, 2018). Cannabis comprises over 500 constituents, among them about 104 cannabinoids have currently been detected (Lafaye et al., 2017). Moreover, two constituents of cannabinoids including cannabidiol (CBD) and delta-9-tetrahydrocannabinol (Δ9-THC) has widely been studied for investigating their pharmacological properties (Lafaye et al., 2017). Medical cannabis has extensively been considered as one of the prospective alternative approaches for the treatments of dementia (Liu et al., 2015; Broers et al., 2019).

Numerous research suggested promising effects of cannabis for decreasing pain and noxious protein from the brain as well as restore cognitive dysfunctions of AD (Esposito et al., 2006a; Russo, 2008; Cheng et al., 2014). Moreover, endocannabinoid signaling has broadly been revealed to control the foremost pathological processes in neurodegenerative disorders, such as misfolding of protein, mitochondrial dysfunction, oxidative stress, excitotoxicity, and neuroinflammation. In this review, we highlight the emerging studies regarding the effect of cannabinoid compounds for treating pain and related neuropathological changes in AD.

## Cannabis Plant

**Figure 1:**
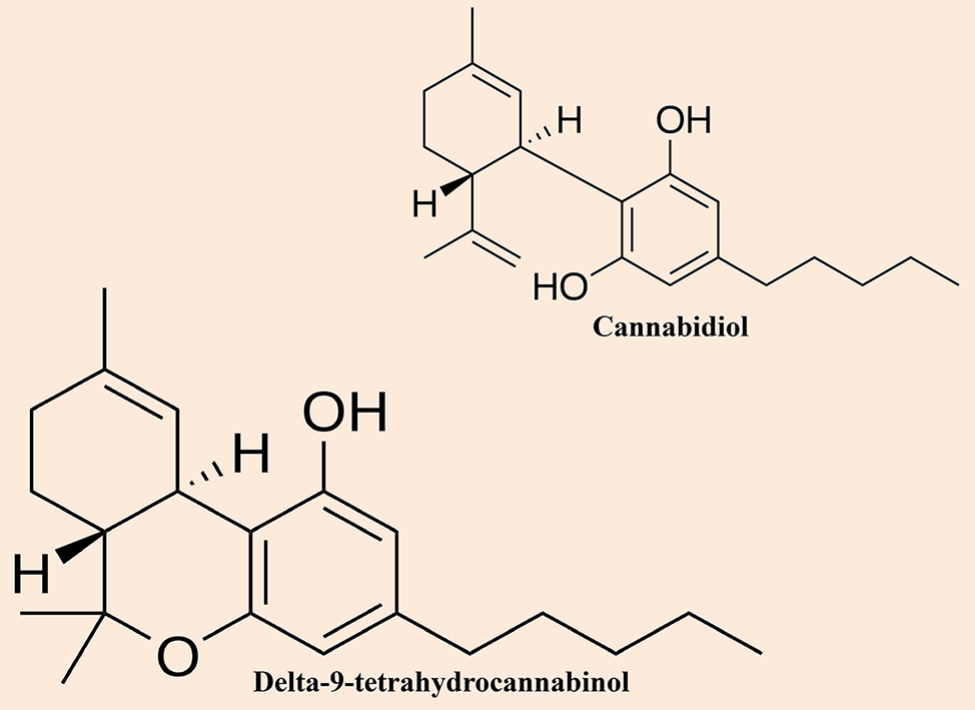
Chemical structures of the most notable cannabinoids (i.e. cannabidiol and delta-9-tetrahydrocannabinol) found in cannabis.

Although cannabis has widely been cultivated and used by mankind for at least 6000 years, (Li, 1973) however, our insight into its pharmacological properties is based on researches that have occurred merely since the end of the 19^th^ century. Cannabinol was the first compound that had been separated in pure form from the cannabis plant (Wood et al., 1899). Primarily, it was mistakenly supposed to be the chief active compound of the cannabis plant that was accountable for its psychoactive actions (Mechoulam and Hanuš, 2000). Furthermore, CBD (**Figure 1**) was the second compound that had been observed by Mechoulam and Shvo (Mechoulam and Shvo, 1963). Subsequently, Gaoni and Mechoulam separated the chief active compound, Δ9-THC (**Figure 1**) in 1964 (Gaoni and Mechoulam, 1964).

There are two main subspecies of the cannabis plant, including *Cannabis sativa* and *Cannabis indica*, and they could be distinguished by their diverse physical properties. Moreover, *indica*-dominant species are small plants with wide, dark green leaves as well as have a higher concentration of CBD than the *sativa* plants where THC content is higher. Conversely, *sativa-*dominant species are usually tall plants and thin with finger-like leaves with a pale green color. *Cannabis sativa* is the favorite choice by consumers because of its higher THC content. The four major compounds are CBD, cannabinol, Δ-8-THC, and Δ-9-THC, which have extensively been studied (Pertwee, 1997; Pertwee, 2008; Pamplona and Takahashi, 2012). Initially, it was believed that CBD was the metabolic parent to Δ-9-THC, however, later it was observed that its biosynthesis occurred by a genetically determined ratio (Russo and Guy, 2006). Although, all four compounds have similar chemical structures, however, their pharmacological effects are different. CBD and Δ-9-THC are the most studied compounds of the cannabis plant.

## Pain, Cognitive Impairment, and AD

The pain sensation is connected with the triggering of the receptors in the primary afferent fibers including myelinated Aσ-fiber and unmyelinated C-fiber (Yam et al., 2018; Uddin et al., 2020k). Furthermore, both nociceptors are initiated when there is a probable toxic stimulus as well as stay silent during homeostasis where the pain is absent. The perception of a sequence of sensory actions is needed for the brain with the purpose of detecting pain as well as generate a response on the way to the threat. Moreover, the perception of pain usually contains three main phases. The first phase is the sensitivity of pain, after that the second phase in which the signals are transferred to the dorsal horn in the spinal cord from the periphery. Finally, the third phase is to execute the transference of the signals to the higher brain through the central nervous system (CNS) (Yam et al., 2018). Numerous research found that chronic pain is connected with the raised objective as well as self-reported cognitive dysfunctions (Cha et al., 2017; Whitlock et al., 2017). These cognitive dysfunctions are not precise to a specific pain modality and could be found in postherpetic neuralgia (Pickering et al., 2014), chronic back pain (Baker et al., 2018) and fibromyalgia (Leavitt and Katz, 2015).

**Figure 2:**
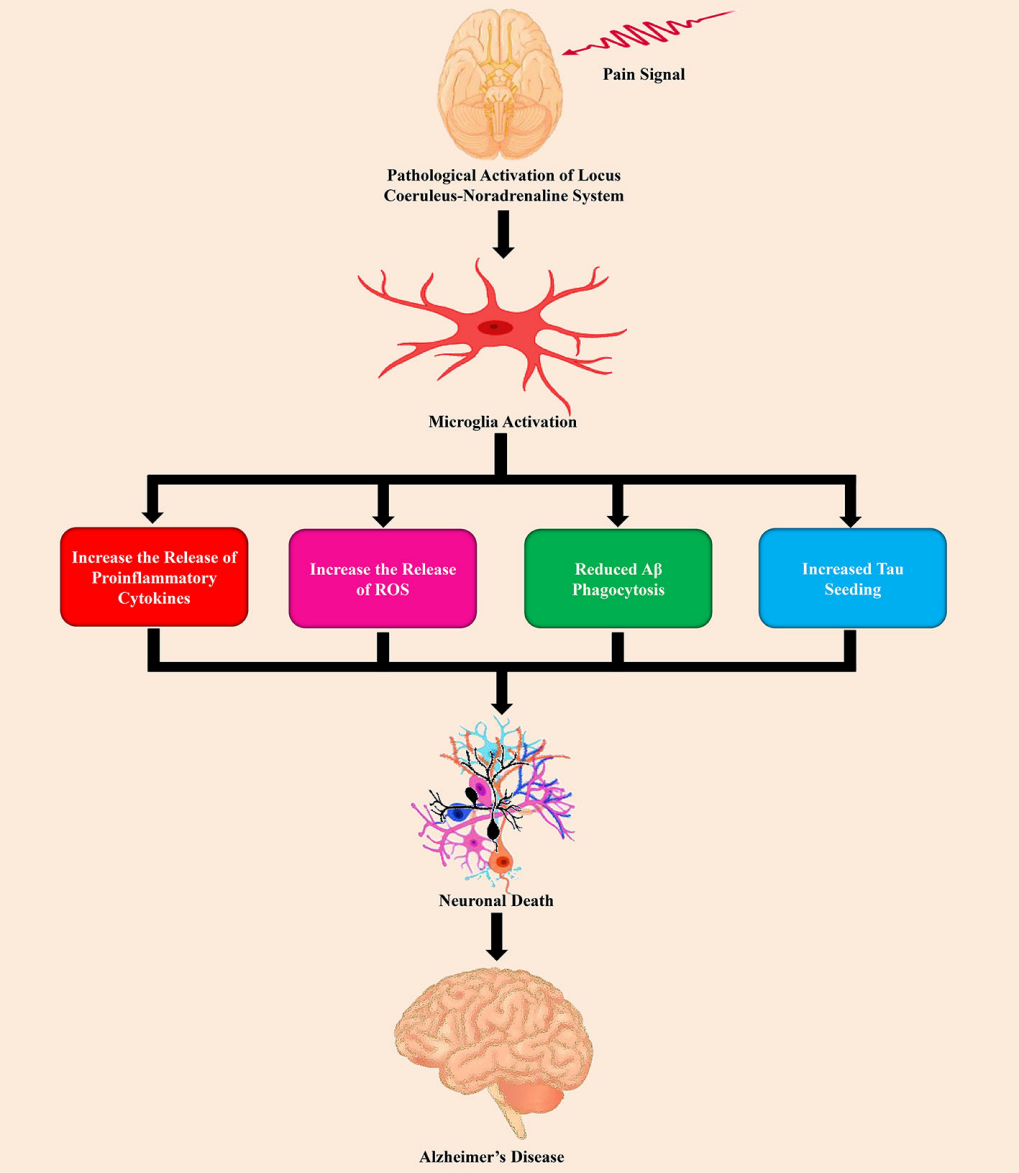
The role of pain stimuli in the pathogenesis of Alzheimer’s disease by the dysfunction of the LC-NE system.

Based on the Einstein Aging Study, Ezzati et al. (2019) assessed the connection of pain intensity, as well as pain interference with incident dementia in 1,114 participants who were 70 years of age or older and 10% of the participants, developed dementia over 4.4 years. In this study, it has been observed that higher levels of pain interference are directly connected with a higher possibility of developing dementia (Ezzati et al., 2019). In a study by Ikram et al. (2019) also advocated that pain interference was considerably linked with AD and related dementia (ADRD). Furthermore, according to the study of Malfliet et al. (2017) chronic pain and AD demonstrated aberrations of the volume of gray matter, and neuroimaging recommended that the patients of cognitive dysfunctions with chronic pain might be connected with alterations of the volume of gray matter in the brain. Importantly, many of these changed brain regions are playing a crucial role in sensory perception, the affective element of cognition and pain (Baliki and Apkarian, 2015; Ng et al., 2017). For example, gray matter volume loss has extensively been observed in the thalamus, parahippocampal gyrus, amygdala, entorhinal cortex, insula, and anterior cingulate cortex (Busatto et al., 2008; Yi et al., 2016; Kang et al., 2019). Numerous studies found that the dysfunction of LC-norepinephrine (NE) system was connected with chronic pain (Cao et al., 2019) as shown in **Figure 2**.

In the CNS, microglia is the main innate immune cells (Ibrahim et al., 2020; Uddin et al., 2020f). Neuroinflammation mediated by microglia is a typical feature in chronic pain (Chen et al., 2018). Proinflammatory microglia release chemokines and cytokines related to inflammation, including umor necrosis factor-α (TNF-α), interleukin (IL)-6, and IL-1β in chronic pain states (Liu et al., 2017; Barcelon et al., 2019). Subsequently, this proinflammatory (**Figure 2**) state contributes to altering the connection of the brain, network function, and synaptic remodeling (Inoue and Tsuda, 2018). Copious studies reported that activation of microglia by persistent exposure to Aβ causes proinflammatory response (Meda et al., 1995; Gold and El Khoury, 2015) that leads to secretion of cytokines, and chemokines, as well as reactive oxygen/nitrogen species (Wyss-Coray, 2006; Rivest, 2009; Balducci and Forloni, 2018). On the other hand, misfolded tau, and truncated tau as well as hyperphosphorylated tau accompany with the proliferation of microglia and amplified the expression of the inflammatory genes (Andrés-Benito et al., 2017). Furthermore, in the brain, reactive microglia causes tau pathology and contribute to the spreading of pathological tau (Nicole et al., 2015). AD brains and chronic pain both show aberrant LC structure and function as well as dynamic alterations in the turnover of NE in LC-projecting regions (Gannon et al., 2015; Llorca-Torralba et al., 2016). In these two disease states, the alterations of NE content might not completely overlap in all brain regions, however, pathological alterations in LC-NE in selective areas can be one of the initiators that are responsible for the dysfunction of the neuron and proinflammatory activation of microglia. Additionally, chronic pain might exacerbate the neuropathogenesis of AD *via* LC-NE-mediated microglial neuroinflammation (Cao et al., 2019).

## Cannabinoids and Pain Regulation in AD

Numerous studies in AD patients have reported reduced, raised, or typical sensory, affective, as well as behavioral reactions to painful stimuli (Benedetti et al., 2004; Pickering et al., 2006; Kunz et al., 2007; Kunz et al., 2009). The duration of impairment to the medial (affective) and lateral (sensory) pain network is recognized in AD. Besides, the locus, intensity, as well as pain quality are controlled by the lateral pain system that intervenes acute or quick sensations of pain. Some studies have recommended that the lateral system is less affected during AD (Scherder et al., 2003; Scherder et al., 2005). On the other hand, the medial pain system interferes the unfavorable, affective reaction to toxic stimuli as well as the neurodegenerative alterations in AD influence the medial pain system during disease (Vogt et al., 1990; Scherder and Bouma, 1997; Scherder and Bouma, 2000).

Cannabinoid receptor 1 (*CNR1*) gene is responsible for encoding the cannabinoid receptor type 1 (CB1R) and this receptor comprises of 472 amino acids in humans as well as 473 amino acids in mouse and rat, with the identification of 97%–99% amino acid sequence amid these species. Numerous research found that some variations of *CNR1* had been connected with the dependence of *Cannabis* (Agrawal and Lynskey, 2009; Hartman et al., 2009; Schacht et al., 2012). Conversely, the cannabinoid receptor 2 (*CNR2*) gene is accountable for encoding the cannabinoid receptor type 2 (CB2R) and this receptor comprises of 360 amino acids in humans. At the protein level, CB2R shares merely 44% sequence homology in comparison with CB1R. Furthermore, the CB2R has also bigger species variations among rodents and humans when compared to CB1R, as the homology of the amino acid sequence is slightly more than 80% between rodents and humans (Liu et al., 2009; Zhang et al., 2015). Two polymorphisms of the CB2R have also been identified in humans (Liu et al., 2009). CB1Rs are not only expressed in the brain, predominantly in the limbic system, cerebellum, substantia nigra, hippocampus, and basal ganglia, but also they are expressed in the peripheral nervous system (PNS), including uterus, bones, liver, testicular tissue, and thyroid (Pagotto et al., 2006; Pertwee, 2006; Russo and Guy, 2006). On the other hand, CB2Rs are mainly expressed in the gastrointestinal system spleen, and immune cells, and slightly in the brain and PNS (Izzo, 2004; Pertwee, 2006). Remarkably, both CB1Rs and CB2Rs are observed in the human placenta and play a pivotal role in controlling the serotonin transporter activity (Kenney et al., 1999).

The characterization of CB_1_ and CB_2_ receptors endorsed the revealing of endocannabinoids (Aso and Ferrer, 2014). In preclinical and clinical studies found that central as well as peripherally situated CB1R had widely been connected with nociception (Manzanares et al., 2006). CB2R are mostly found in the cells of the immune system might play an essential role in reducing pain, since they have extensively been linked with the inhibition of pain and inflammatory processes (Ashton and Glass, 2007). Furthermore, the endocannabinoid system (ECS) has widely been connected with central stress-mediated analgesia (Hohmann et al., 2005). THC has also revealed anti-inflammatory effects in many preclinical and clinical studies. THC has also a great effect on serotonergic, glutamatergic, and opioid receptors that have played a pivotal role in the development as well as regulation of neuropathic pain (Manzanares et al., 2006). Therefore, these results recommend that although endogenous cannabinoids might be necessary for the homeostatic regulation of pain, however, exogenous cannabinoids, including synthetic cannabinoids and THC might be a promising adjunct therapy for the treatment of clinical pain (Fine and Rosenfeld, 2013).

Pain regulation is the initial medical uses of cannabinoids. Many studies have reported that the analgesic effects of cannabinoids in diverse kinds of pain, such as mechanical, heat, and chemical pain, and they also reduced inflammation as well as neuropathic pain (Fine and Rosenfeld, 2013; Donvito et al., 2018). The ECS is also played a crucial role in the control of nociception (Pacher et al., 2006). Similarly, endocannabinoids have also a great effect on the regulation of inflammation as well as neuropathic pain (Donvito et al., 2018). Apart from the CB1R, there is also positive evidence for advocating the involvement of the transient receptor potential vanilloid-1 (TRPV1) and CB2R in cannabinoid-induced control of pain (Jhaveri et al., 2007; Akopian et al., 2009). Moreover, scientists are now focusing the phytocannabinoids for the management of nociception as well as other neurological complications. For example, CBD has widely been reported to control chronic pain in many studies (Donvito et al., 2018).

In a clinical setting, pain could be a highly subjective measure. It is very challenging to measure and manage pain in dementia patients because the communication is frequently reduced as well as many other symptoms present concurrently (Chow et al., 2016). Nowadays various analgesics including nonsteroidal anti-inflammatory drugs (NSAIDs), opioids, and acetaminophen are used for treating pain in dementia (Sherman et al., 2018). Conversely, as NSAIDs must use carefully as they exert gastrointestinal side effects and the use of opioids should be carefully monitored because of common adverse effects including nausea, vomiting, and sedation (Sherman et al., 2018). Furthermore, targeting the ECS has demonstrated favorable effects for the management of pain in diseases including multiple sclerosis and fibromyalgia (Svendsen et al., 2004; Tsang and Giudice, 2016). Recently, nabilone, a synthetic cannabinoid has used in AD patients for examining pain as an exploratory finding of the clinical trial (NCT02351882) by using the pain assessment in advanced dementia scale (ClinicalTrials.gov, 2020). Based on the cautious assessment of the effects of the ECS as well as preclinical studies, it can be said that cannabinoids might show a positive effect on pain in AD (ClinicalTrials.gov, 2020). Therefore, the use of randomized controlled trials is needed to assess the safety as well as the efficacy of cannabinoid for treating pain as a primary finding in the dementia patients.

## Endogenous Cannabinoid System in Alzheimer’s Brains

The investigation of human post-mortem samples exposed several changes in the composition of ECS as well as signaling in AD brains, though the alterations in the pathophysiology of the disease remain unclear until now. Likewise, the alterations in the expression of CB1R in AD are unknown. Although, some studies found that a considerable decrease in the levels of CB1R in cortical areas as well as in neurons faraway from senile plaques (Ramírez et al., 2005; Solas et al., 2013), however, many investigations have reported no changes in the distribution and expression of CB1R in hippocampus and cortex in AD (Benito et al., 2003; Ahmad et al., 2014; Lee et al., 2010; Mulder et al., 2011). Moreover, no relationship between the levels of CB1R and any pathological marker of AD has been observed (Solas et al., 2013). On the other hand, the significant levels of CB2R have found in AD brains because of the expression of CB2R on microglia nearby senile plaques (Ramírez et al., 2005; Solas et al., 2013). Importantly, the expression of CB2R levels connects with the levels of Aβ_42_ and the accumulation of plaque, even though not with cognitive impairment (Solas et al., 2013), recommending that these pathogenic events trigger the expression of CB2R. Furthermore, both CB1R and CB2R in the brain of AD are nitrosylated, as well as this can lead to the impaired connection of these receptors to downstream effector signaling molecules (Ramírez et al., 2005).

Some investigations demonstrated other elements of ECS in AD human samples. The initial study examining the levels of endocannabinoid showed no changes between healthy controls and AD patients in the plasma concentrations of 2-arachidonoyl-sn-glycerol (2-AG) and anandamide (AEA) (Koppel et al., 2009). However, in a study by Jung et al. (2012) reported that the lower levels of AEA in temporal cortices and midfrontal in AD when compared to control subjects in post-mortem brain samples, which reciprocally connected with the levels of Aβ_42_ in the neurotoxic brain as well as cognitive deficiencies documented in these patients, recommending an involvement in the Aβ_42_-dependent impairment of AEA to cognitive decline. Besides, several changes have widely been observed in the activity of the enzymes associated with the synthesis of endocannabinoid as well as degradation in the brains of AD. Therefore, fatty acid amide hydrolase (FAAH), the endocannabinoid metabolizing enzyme, is up-regulated in AD brains both in peripheral blood mononuclear cells (D’Addario et al., 2012) and neuritic plaque-related glia (Benito et al., 2003), and this can lead to the increase of the degradation of AEA in the surrounding area of the senile plaque. Furthermore, the increased expression of FAAH might have two detrimental outcomes in the progression of disease such as limitation of the availability of neuronal AEA and increases proinflammatory molecules mediated by the metabolites of AEA including arachidonic acid (Calder, 2005). In a study by Mulder et al. (2011) reported that changed 2-AG signaling throughout late phases of AD because of the combination of impaired recruitment of monoacylglycerol lipase (MAGL) as well as raised levels of diacylglycerol lipase that promote synapse silencing in AD.

## Effect of Cannabinoids on Alzheimer’s Hallmarks

### Aβ Pathology

As stated earlier, atypical production and accumulation of Aβ peptides in the brain are considered as the hallmark of AD (Al Mamun et al., 2020a; Sharma et al., 2020; Uddin et al., 2020i). Exogenous cannabinoids, a neuroprotective agent, have persistently been disclosed to restrain memory deficits in Aβ-treated animal models for both synthetic selective cannabinoid receptors agonists (Haghani et al., 2012; Wang et al., 2013), as well as mixed cannabinoid receptors agonists (Ramírez et al., 2005; Martín-Moreno et al., 2011; Fakhfouri et al., 2012) and natural CBD (Martín-Moreno et al., 2011). For example, prolonged treatment of two separate transgenic (Tg) mice models of brain amyloidosis with CB1R agonist arachidonyl-2-chloroethylamide (ACEA) (Aso et al., 2012), CB2R agonist JWH-133 (Martín-Moreno et al., 2012; Aso et al., 2013), or non-selective agonist WIN55,212-2 (Martín-Moreno et al., 2012) resulted in the improvement of cognitive parameters. Interestingly, the protective effects of cannabinoid compounds in Tg animals against cognitive deterioration had declined with disease advancement (Aso et al., 2012; Aso et al., 2013).

**Figure 3:**
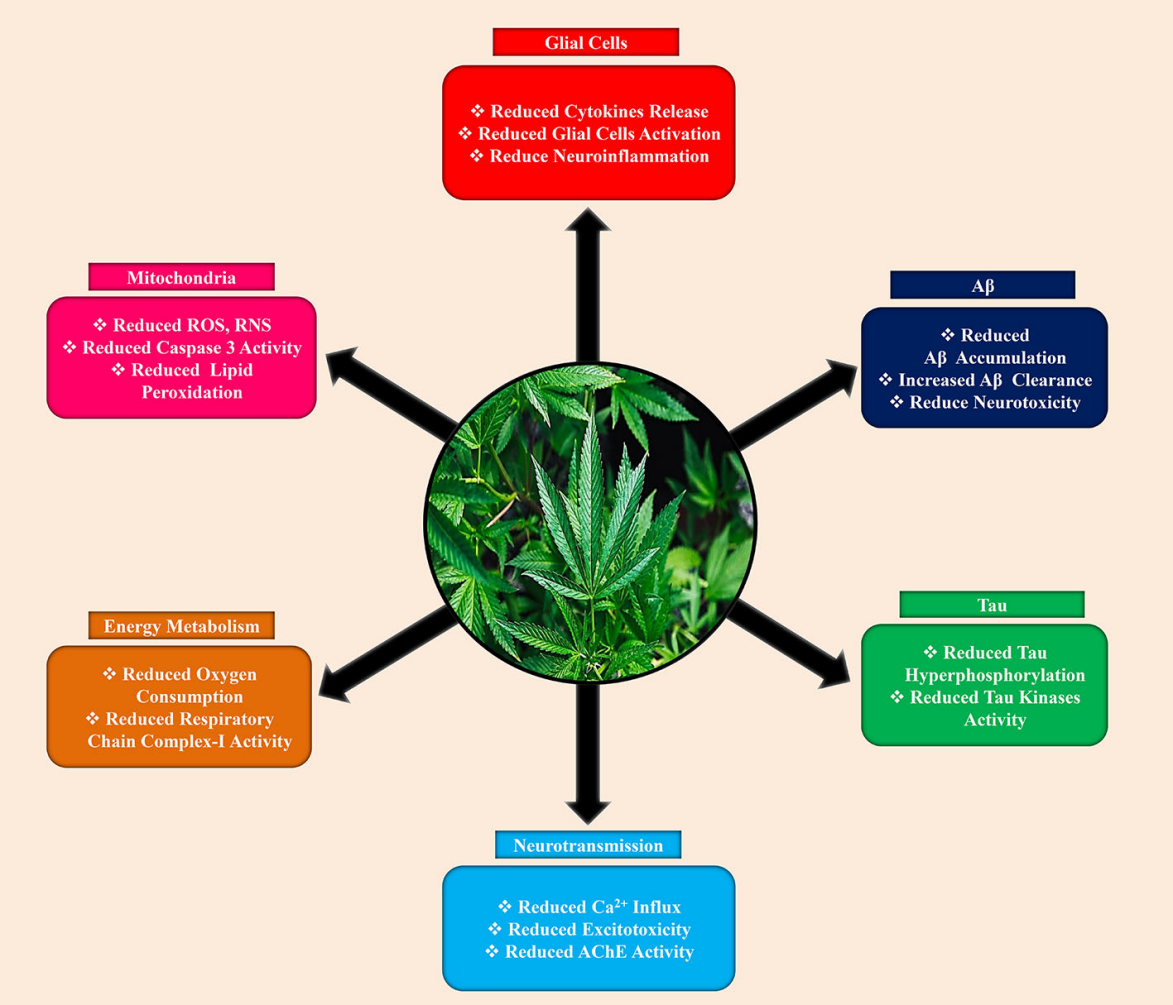
Neuroprotective effects of cannabinoids against Alzheimer’s disease.

The neuroprotective mechanisms of cannabinoid that eventually responsible for memory improvement in Aβ are multiplex and are presumed to take action in parallel or interacting within them. Even if most of the suggested defensive mechanisms of action depending on the magnitude of cannabinoids to indirectly alleviate the devastating effects of Aβ, the direct consequences of cannabinoids on Aβ processing also proposed. For that reason, activation of CB2R provoked Aβ clearance by human macrophages (Gangaidzo et al., 1997; Wu et al., 2013) and supported Aβ transfer through the choroid plexus (Martín-Moreno et al., 2012). This supports the Aβ-clearance across the blood-brain barrier (BBB) had also revealed for the 2-AG, a synthetic endocannabinoid that CB1R/CB2R agonist, in *in vitro* and *in vivo* BBB clearance models (Bachmeier et al., 2013). In the Tg AD mice model, these results could interpret that prolonged administered with CB2R or CB1R/CB2R agonists markedly reduced Aβ levels (Martín-Moreno et al., 2012). Conversely, chronically treated with ACEA (Aso et al., 2012) or HU-210 (Chen et al., 2010) were observed no significant effects on Aβ generation, accumulation, or clearance in Tg AD animal models. Nevertheless, the Stumm et al. (2013) demonstrated that APP23/CB1(-/-) mice decreased the formation of Aβ plaque and APP levels, probably caused by a shift in intracellular APP transport, even though the experimental mice showed elevated cognitive deficits. Furthermore, Chen et al. (2013) study disclosed that Δ-9-THC, one of the cannabinoid isomers, markedly elevated the neprilysin (an enzyme that can degrade Aβ) expression, but not β-secretase (BACE1), as a result to a significant decreased of Aβ plaques in 5xFAD APP Tg animal model. This study fizzled to elucidate, the distinctive CB1R or CB2R function in such a Δ-9-THC effect on Aβ-clearance (**Figure 3**).

### Tau Pathology

Hyperphosphorylation and aggregation of tau is another major hallmarks of AD (Uddin et al., 2020h: Uddin et al., 2020g). Accumulating evidence suggested that cannabinoids also play a significant role in tau pathology. Previously, Aβ-induced PC12 neuronal cell culture study revealed that CBD, ACEA, and WIN55,212-2 impede hyperphosphorylation of tau protein (Esposito et al., 2006a). For CBD, this effect might mediate by reducing of phosphorylated glycogen synthase kinase-3β (GSK-3β) (**Figure 3**), as a consequence activation of the Wnt/β-catenin signaling pathway that eventually responsible for the reduction of neuronal apoptosis (Ferrer et al., 2005; Esposito et al., 2006a). On the other side, the effect of both the selective (ACEA) and non-selective (WIN55,212-2) CB1R agonist on tau hyper-phosphorylation was selectively induced by the CB1R in Aβ-induced C6 glioma cells co-cultured with PC12 neuronal cells through down-regulating inducible nitric oxide synthase (iNOS) and nitric oxide (NO) generation (Esposito et al., 2006b). In line with the molecular mechanism of the CB1 receptor on tau hyper-phosphorylation, chronically treated APP/PS1 mice with ACEA decreased the proportion of phosphorylated tau at Thr181 area (a site nearby Aβ plaques), perhaps ACEA-mediated reduction in GSK-3β detrimental effects (Aso et al., 2012). Additionally, a particular mechanism for the CB2R in the regulation of tau phosphorylation has also proposed. For example, in double Tg mice, chronically administered specific CB2R agonist (JWH-133) lowered tau hyper-phosphorylation in the surrounding of Aβ plaques that may be achievable by reducing the action of GSK-3β, p38, and stress-activated protein kinase/c-Jun N-terminal kinase (SAPK/JNK) (Aso et al., 2013).

To validate these trials, recently, one study reported that long-term treatment with Sativex^®^, an approved medicine that made by mixed Δ-9-THC and CBD natural extracts, significantly decreased NFTs in PK(-/-)/Tau(VLW) (parkin-null, human tau overexpression) mice model (Casarejos et al., 2013). The investigators of this study proposed cannabinoid strengthen of autophagy improving redox status as likely mechanisms responsible for the lowering of tau accumulation.

## Effect of Cannabinoids Against Neuroinflammation in AD

Neuroinflammation, primarily expressed as microglial activation, is an important characteristic in AD that accelerates cell damage and neuronal loss as well (Akiyama et al., 2000; Hensley, 2010; Sardi et al., 2011; Uddin et al., 2020c; Uddin et al., 2020f). Accumulating data indicating that CB2R is fundamentally responsible for several immune reactions, where they are capable of suppressing microglia-induced neurotoxicity, eventually, cannabinoids compounds that act on the CB2R can serve as an anti-inflammatory agent (**Figure 3**) in neuroinflammation (Cabral and Griffin-Thomas, 2009). Previously, it has demonstrated that by activating CB2R notably decreased Aβ-mediated neuroinflammatory response in several AD animal models. For instance, several studies reported that the microglial response and production of proinflammatory mediators were significantly reduced by both the selective or mixed CB2R agonists in Aβ-induced animal brains (Ramírez et al., 2005; van der Stelt et al., 2006; Esposito et al., 2007; Fakhfouri et al., 2012; Wu et al., 2013). Likewise, in APP Tg models, the proportion of reactive microglial cells adjacent to the Aβ deposition area and concentration of the proinflammatory cytokines were depleted by selective CB2R agonists (Martín-Moreno et al., 2012; Aso et al., 2013). Furthermore, in a tauopathy animal model, Sativex^®^ can be able to dampen the microglial activation (Casarejos et al., 2013), though no directly implicating proof of CB2R or other receptors in such effects provided. Moreover, recently one study reported that chronically treated with CB1R agonist ACEA decreased astrocytic reactivation and lower expression of interferon-γ in AβPP/PS1 Tg mice (Aso et al., 2012). Surprisingly, CBD had not shown an affinity to CB1 or CB2 receptors but manifested anti-inflammatory characteristics in the AD animal model (Esposito et al., 2006a; Martín-Moreno et al., 2011). The exact location where CBD expresses its neuroprotective effects is yet to confirm, but few findings indicate that CBD may have selective interaction with peroxisome proliferator-activated receptors-γ (PPARγ) (Esposito et al., 2011).

In AD, the enzymes that are related to AEA and 2-AG deterioration may also responsible for regulating the inflammation. FAAH is an enzyme that manifested not only in the neurons but also astrocytes, where it can contribute to a role in response to the inflammatory process. A study reported that FAAH overexpressed in astrocyte and notably sustained in the neuroinflammatory process, which was possessed to assist the detrimental process mediated by toxic insults due to the lowering of endocannabinoid tone (Benito et al., 2003). However, FAAH-knockout mice expressed more responsive to Aβ than wildtype astrocytes and exhibited higher proinflammatory phenotype, distinguished by an elevation in cytokine production as well as cell death likely due to the alternation of signaling pathways involved in cell survival and inflammation, including, extracellular signal-regulated protein kinases 1/2 (ERK1/2), p38 mitogen-activated protein kinase (p38MAPK), and nuclear factor kappa-light-chain-enhancer of activated B cells (NF-κB), as well as to the escalation in inflammatory molecules such as iNOS and cyclooxygenase (Benito et al., 2012). The researchers of this study disclosed that these processes not related to CB1 or CB2 receptors but PPAR-α, PPAR-γ, and TRPV1. So far, in astrocytes, the proinflammatory phenotype could not be initiated by the pharmacological blockade of FAAH, suggesting that the audited effects in astrocytes absent FAAH could be due to compensative shifts that result from the probably prolonged augment of N-acylethanolamines. The result of this study indicates that an exceedingly long-term prolongation of endocannabinoid tone may have detrimental effects. On the contrary, the inhibition of MAGL, an enzyme that role in hydrolyzing endocannabinoids (Nomura et al., 2011), and regulate the arachidonic acid release, reduced the Aβ levels and diminished neuroinflammation in AD animal model (Piro et al., 2012). These findings were verified by the pharmacological MAGL inhibitor, which reiterated the proinflammatory cytokine-reducing effects through lowering prostaglandin generation, rather than intensified endocannabinoid signaling pathway.

## Effect of Cannabinoids Against Oxidative Stress in AD

Overwhelming evidence demonstrated that mitochondrial dysfunction as a causative factor to neurodegenerative diseases, including AD (Ferrer, 2009; Ankarcrona et al., 2010; Burchell et al., 2010; Uddin and Kabir, 2019). Impaired mitochondrial function emerges at the early stage in AD, which eventually causative factor for neurons exhaustion as a consequence of converging in the reduction of energy generation, elevated energy demand, and uncontrolled oxidative stress (Ferrer, 2009; Uddin et al., 2020b). The cannabis derivatives also have a potent antioxidant property, particularly CBD was shown more protective than α-tocopherol against glutamate neurotoxicity (Hampson et al., 1998). Besides, CBD not only suppressed ROS generation and lipid peroxidation but also reduced caspase 3, (**Figure 3**) and intracellular calcium levels in Aβ-induced PC12 neuronal cells (Iuvone et al., 2004). Furthermore, it also lowered iNOS and NO in similar conditions (Esposito et al., 2006b). Moreover, other cannabinoids, for example, selective CB2R agonists exerted antioxidant properties in AD animal models. Therefore, JWH-133, a selective CB2R agonist, lowered hydroxynonenal adducts (produced from lipid peroxidation), elevated superoxide dismutases-1 (SOD-1) and superoxide dismutases-2 (SOD-2) in surrounding of plaques in APP/PS1 mice, suggesting the role of CB2R in lowering harmful effects against oxidative stress (Aso et al., 2013). Chronically administered with Sativex^®^ in the tauopathy animal model was also proposed to lower the free radicals and mitochondrial activity in the tauopathy animal model.

## Effect of Cannabinoids on Energy Metabolism in AD

The functional role of cannabinoid receptors in regulating neuronal energy metabolism has been becoming great attention in the scientific community. However, only a few studies available so far to confirm the direct possession of CB1R over neuronal respiration and energy generation. For instance, Bénard et al. (2012) used anti-CB1R antibodies, disclosed the protein localization of CB1R nearly 30 percent of neuronal mitochondria, which when triggered by exogenous/endogenous cannabinoids lowers the respiratory chain complex-I activity and oxygen consumption, probably *via* cyclic adenosine monophosphate (cAMP) and protein kinase-A (PKA) signaling. These results are supported by Athanasiou et al. (2007) findings, which reveal that all of the partial CB1R agonists including AEA, Δ-9-THC, and HU-210 markedly reduced oxygen consumption (**Figure 3**) and mitochondrial membrane potential. However, care must be taken to interpret these findings due to using commercial anti-CB1R antibodies (Morozov et al., 2013).

## Effect of Cannabinoids on the Modulation of Neurotransmission in AD

In recent years, acetylcholine esterase (AChE) inhibitors mostly approved by drug administration to treat AD, which rises the acetylcholine (ACh) availability to some extent alleviating this neurotransmitter insufficiency in AD patients, or they are non-competitive N-methyl-D-aspartate (NMDA) receptor antagonists, which blocks the NMDA-associated ion channel consequently lower calcium influx and restrain excitotoxicity (Kabir et al., 2019b; Kabir et al., 2019a; Kabir et al., 2020b). Surprisingly, particular cannabinoid molecules play a role in the same target compared to contemporary medicaments, resulting in analogous or increased favorable effects. For example, Δ-9-THC competitively impedes AChE, (**Figure 3**) consequently elevating ACh levels, as well as hindering AChE-mediated Aβ deposition by binding in the peripheral-anionic-site of AChE, the dreadful area engaged with amyloidogenesis (Eubanks et al., 2006). Some synthetic cannabinoids can act as stereoselective NMDA receptors blockers (Feigenbaum et al., 1989). For example, HU-211, which can protect cells against NMDA-inducing neurotoxicity (Feigenbaum et al., 1989; Eshhar et al., 1993; Nadler et al., 1993). The neuroprotective activity of HU-211 caused by direct binding to NMDA receptors, unfortunately not to cannabinoid receptors, however; the widely considered cannabinoid-induced neuroprotective effects against excitotoxicity may be accomplished through several mechanisms, including suppression of presynaptic glutamate release (Marsicano et al., 2003), interruption of voltage-dependent calcium channels (Mackie and Hille, 1992), and the prohibition of calcium release (Zhuang et al., 2005), which predominantly indicates the direct or indirect involvement of CB1R.

## Conclusion

Cannabinoids act by targeting several signaling processes, such as pain, abnormal processing of Aβ and tau, neuroinflammation, excitotoxicity, oxidative stress, and mitochondrial dysfunction, which play a pivotal role in the management of AD. Cannabinoids also ameliorate behavioral and cognitive dysfunctions. Therefore, due to these extensive medical uses of cannabinoid compounds, it can be said that targeting the endocannabinoid system can be a promising strategy to develop an effective therapy for the management of AD. Furthermore, cannabinoids may demonstrate a safe and reliable low-cost therapy, with limited side effects. Future research is needed to investigate the use of cannabinoids for the treatment of AD in a clinical trial setting.

## Author Contributions

MU conceived the original idea and designed the outlines of the study. MU, AM, and DS wrote the draft of the manuscript. MU prepared the figures for the manuscript. GA, AP, SB, SM, HE-S, MB-J, and MA-D revised and improved the draft. All authors contributed to the article and approved the submitted version.

## Conflict of Interest

The authors declare that the research was conducted in the absence of any commercial or financial relationships that could be construed as a potential conflict of interest.

## Abbreviations

AD, Alzheimer’s disease; Aβ, amyloid beta; CBD, cannabidiol; ECS, endocannabinoid system; LC, locus coeruleus; NE, norepinephrine; NFTs, neurofibrillary tangles; Δ9-THC, delta-9-tetrahydrocannabinol.
